# Prevalence of vernal keratoconjunctivitis and its associated factors among children in Gambella town, southwest Ethiopia, June 2018

**DOI:** 10.1371/journal.pone.0215528

**Published:** 2019-04-18

**Authors:** Abiy Maru Alemayehu, Betelhem Temesgen Yibekal, Sofonias Addis Fekadu

**Affiliations:** Department of Optometry, College of Medicine and Health Sciences, University of Gondar, Gondar, Ethiopia; Aston University School of Life and Health Sciences, UNITED KINGDOM

## Abstract

**Introduction:**

Vernal keratoconjunctivitis is a chronic bilateral severe form of allergic conjunctivitis which affects normal activities in school/work. It is a severe form of allergies in warm and dry tropical and sub-tropical countries. Its prevalence in Ethiopia ranges from 5.2% to7.3%. Most studies are institution based and do not address specific factors associated with vernal keratoconjunctivitis. There is no a study that shows the magnitude of vernal keratoconjunctivitis in the study area.

**Objective:**

To assess the prevalence of vernal keratoconjunctivitis and its associated factors among children in Gambella town, Southwest Ethiopia, 2018.

**Methods and materials:**

A community based cross-sectional study was conducted from April 25 to May 12, 2018, in Gambella town. A total of 578 study participants were selected using a systematic random sampling technique. A pre-tested semi-structured questionnaire, torch, and magnifying loop were used to collect data. The data was entered into epidemiological information 7.1 and exported to statistical package for social science for analysis. Binary logistic regression analysis model was fitted to identify factors associated with vernal keratoconjunctivitis. Odds ratio with respected 95% CI was used to identify the direction and strength of association.

**Results:**

A total of 574 children participated in this study representing a response rate of 99.30%. The mean age of the participants was 9.74±4.0 years. The prevalence of vernal keratoconjunctivitis was 11.10% (95% CI: 8.70, 13.90). Male sex (adjusted odds ratio = 4.12(95% CI: 1.42, 11.91)), close animal contact (adjusted odds ratio = 3.45(95% CI: 1.14, 10.41)), dust exposure (adjusted odds ratio = 3.38(95% CI: 1.31, 10.04)), and personal systemic allergy history (adjusted odds ratio = 4.82(1.40, 16.72) were independently associated with vernal keratoconjunctivitis.

**Conclusion:**

The prevalence of VKC was high among children in Gambella town. Sex being male, close animal contact, personal systemic allergy history, and dust exposure were positively associated with vernal keratoconjunctivitis independently.

## Introduction

Allergic conjunctivitis is the inflammation of the conjunctiva (caused by hypersensitivity type I reaction) due to the immune response to the allergens[1]. Vernal (springtime) keratoconjunctivitis (VKC) is a chronic bilateral inflammation of the conjunctiva/cornea which is manifested by the presence of giant/cobblestone papillae at the tarsus/limbus [2–5].

Commonly presenting symptoms of VKC are stringy mucoid discharge, itching swollen eyelid, tearing, burning, red- eye, foreign body sensation, and photophobia. Whereas most common signs of VKC are lid edema, chemosis, tarsal papillae, Horner Trantas-Dots, brownish discoloration of eyeballs, darkened eyelids limbal infiltrates [6–8].

Clinically 3 types of VKC are identified. Limbal type is with a fine gelatinous limbal infraction and Horner Trantas-Dots; the palpebral type has giant papillae > 1mm in diameter on upper tarsus only and mixed type include both types [5,9]. Limbal and mixed VKC is the predominant form in central and southern African whereas, palpebral VKC is most frequent in Europe and America [7,8,10].

VKC makes up 0.1–0.5% of ocular diseases in developed countries. In Europe, the prevalence of VKC ranges from 1.2–10.6 cases per 10000 population[1]. However, its size is higher in warm and dry tropical and sub-tropical countries of Africa, The Middle East, Latin America, and Asia[11]. In Africa, the prevalence of VKC reaches 2–37% [12–15] and in Ethiopia, it accounts for 5.3%-7.3% with male dominance [16–18].

VKC is a major cause of hospital referrals among children in Africa, the Middle East, and Asia. In Africa, VKC is responsible for the highest percentage (21.0%) of general eye clinic attending children and cause of school non-attendance [12,19]. Over a quarter of 2,250 children seen at a tertiary referral pediatrics eye clinic in East Africa had VKC[14,20]. VKC is a severe form of allergic conjunctivitis which affects normal activities in school/work and is a blinding disease which needs high social costs[6,21]. More than one-third (36.4%) of children with VKC had missed school ≥ 1day/month[12,19].

About 23% of patients have a perennial form of VKC and more than 60% have a seasonal recurrence[22]. Numerous patients have an exacerbation of VKC in spring season[1]. In Europe and Asia VKC tends to get worse in winter[23] but all year round in Africa[24].

VKC is a major health problem in dry and hot regions of Africa. Effect of climate, sun exposure[9,25], male gender, economic status, dust and wind exposure, underlying atopy, kerosene/wood fire smoke, and close animal contact are identified associated factors of VKC [4,16,26].

The possible management options of VKC includes from supportive to medical intervention based on its severity. These includes; regular hand and face washing, staying out of the sun, keeping away from dust and smokes[25], and avoiding touching or rubbing of the eyes[27], cold compress, artificial tears, steroidal and non-steroidal drugs with care of duration of treatment to avoid long-term use complications[1,9,10].

In the absence of proper management, 4.6% of VKC patients lead to visual impairment and total visual loss [21]. The severe form of VKC is a potentially blinding disease in developing countries following its worrisome complications including keratitis, shield ulcer, keratoconus, corneal hydrops, astigmatism, cataract and glaucoma[7,9,10,28].

Even if most studies are conducted on VKC, they are hospital/institution based and don’t address different factors associated with it. There are studies done in Rwanda and Ethiopia that showed the prevalence and associated factors of VKC. However, Geographical, hormonal, ethnic and other factors contribute to the different magnitude of VKC [9,29,30] and there are no such studies done in the study area about VKC. The previous study examined patients in Gondar which is one of the high land area in the country so has climate differences compared to Gambella, hence the need to conduct this new study[31]. This study showed the magnitude and associated factors of VKC among children in Gambella town.

## Methods and materials

### Study design and population

The community based cross-sectional study was conducted from April 25 to May 12, 2018, in Gambella town which has a population size of 61,044 of which 16,075 accounts for under 18 years.

### Sample size determination

The sample size was calculated using a single population proportion formula for the proportion of vernal keratoconjunctivitis. To achieve this, the proportion of VKC among children 5.8% [16] was considered for which, the sample size came to 578. The margin of error 2%, the 95% confidence level and 10% for the non—response rate were considered during the sample size calculation.

### Sampling technique and procedures

The study participants were selected using a systematic random sampling technique by assuming that each house contains at least one child. A total number of households were taken from each kebele (smallest administrative units in Ethiopia) administration. By calculating the interval **(k)** using the formula K = N/n, i.e. 10,152/578 = 18

Where, n = sample size, N = total number of households in the town. So, by selecting one house out of 18 households using simple lottery method as a starting point (it was the 4^th^ house) every 18^th^ house was included in the study. When the selected houses were closed by default they were rechecked two times in the next days and if not opened the next houses were included. Next houses were also included if no child at all in the selected house. In a house that contains more than one children lottery method was applied to select one participant per house. All procedures were undertaken after permission is granted from house head who were identified by interviewing in each selected households.

The study was conducted after ethical clearance was obtained from the ethical review board of the University of Gondar and Gambella town administrative office. Written informed consent was taken from each study participants’ parent or guardian. Assent was obtained for those who were aged ≥13 years after explaining the purpose of the study, in addition to the consent of the parent or guardian. Assent was taken for those children 6–12 year and parents’ consent were also taken as well. Besides this, only parents’ consent was taken for those children < 6 years. They were given full right to participate and to refuse or withdraw at any time they want. Confidentiality of the information obtained was assured by avoiding personal identifiers like a name from data collection tool and also through coding and locking the data.

### Operational definitions

**Vernal keratoconjunctivitis**: the presence of conjunctival papillae ≥ 1mm diameter over the upper tarsal plate and/or limbal hypertrophy[32] with itching sensation and/or at least one of the following symptoms in the last 6 months: photophobia, sticky mucous discharge, redness, tearing and foreign body sensation.

**Palpebral Vernal Keratoconjunctivitis (PVKC**): the presence of papillae > 1 mm on the tarsal conjunctiva without limbal involvement with itching sensation and/or at least one of the following symptoms photophobia, sticky mucous discharge, redness, tearing and foreign body sensation in the last 6 months[16,32].

**Limbal Vernal Keratoconjunctivitis (LVKC):** having at least one of the following limbal findings: thickening, broadening, opacification, Horner-Trantas dots with itching sensation and/or at least one of the following symptoms in the last 6 months: photophobia, sticky mucous discharge, redness, tearing and foreign body sensation [30].

**Mixed Vernal Keratoconjunctivitis (MVKC)**: is when features of both limbal and tarsal VKC[5].

**Children**: children between 2–18 years who lives in Gambella town at least for 6 months.

**Animal contact:** family having at least one type of domestic animal and a child plays with or touch the animal/their dander at least once in a day and cause ocular irritation, itching or burning [12].

**Dust exposure**: at least history of one episode of exposure to dust to the eye that resulted to itching foreign body sensation/ ocular irritation in the last 6 months [12].

**The family history of ocular allergy:** any known allergic eye diseases of mother and/or father, brother and/or sister.

**The family history of non-ocular allergy**: any known of the following diseases; asthma, rhinitis, eczema in father and/or mother, brother and/or sister.

**Personal non-ocular allergy**: any known of allergy disease in the body that does not involve the eye mainly.

**Household head:** A person who was in charge of or responsible for the family was considered as household head (report upon asking during data collection period) who gave us the consent to perform the study.

**Income**: is the sum of all the wages, salaries, profits, interest payments, rents, and other forms of earnings received in a given period of time.

### Data collection tools and procedure

The data collection tool (questionnaire) was adopted by reviewing different kinds of literature on similar studies [15,16,26,30]. The pre-tested and semi-structured Amharic version of the questionnaire was used for face to face interview. 3x magnifying loops and ophthalmic torches were used for physical examination.

The data was collected by 2 optometrists and 2 ophthalmic nurses under one supervisor (BSc optometry) after appropriate training was given for two days on how to collect data, the approach to participants and on case identification of vernal keratoconjunctivitis by the principal investigator.

### Data quality assurance

A semi-structured English version of the questionnaire was translated to Amharic version and back to English to ensure accuracy and consistency by language professionals. The pre-test was done out of the study area to validate the questionnaire by taking 5% of the sample size and modifications were done accordingly to ensure the appropriateness and common understanding. Training was given to data collectors on how to diagnose VKC using magnifying loop, torches and different images of conjunctival signs. On the field work, the supervisor has closely followed the day-to-day data collection process and ensure the internal consistency of the collected data. Finally, 5% of the collected samples were checked for completeness by the principal investigator in each day.

### Data processing and analysis

After cleaning and coding, the data were entered into Epi info 7.1 and were exported and analyzed using statistical package of social sciences (SPSS) version 23. The descriptive statistics were summarized using summary statistics such as frequency tables, graphs, percentages, means, and standard deviations.

The variables that were found with P<0.25 at binary logistic regression were entered to multivariable analysis in which multiple logistic regression was used and those variables with a p-value less than 0.05 were considered as statically significant[16].

## Results

### Sociodemographic characteristics of the study participants

A total of 574 study participants were included in the study with a response rate of 99.30%.

The mean age of study participant was 9.74±4.0 years. Forty-four percent (250) of the study participants were in the age group of 11–18 years. More than half (55.6%) of the participants were males. Regarding the religion of the participants, almost half 272(47.4%) of them were protestant. More than half 344(60%) of children were primary school students. Nearly half (47.4%) of household heads finished primary schools level (Table 1).

**Table 1:** Socio-demographic characteristics of study participants among children living in Gambella town, Southwest Ethiopia, 2018 (n = 574).

Variables	Frequency	Percent
Age group		
2–5 years	103	17.9
6–10 years	221	38.5
11–18 years	250	43.6
Sex		
Male	319	55.6
Female	255	44.4
Ethnicity		
South	155	27.0
Oromo	123	21.5
Amhara	115	20
Gambela	112	19.5
Tigray	69	12.0
Religion		
Protestant	272	47.4
Orthodox	161	28.0
Muslim	93	16.2
Catholic	45	7.9
Others	3	0.5
Child educational status		
No school	97	16.9
KG	86	15.0
Primary	344	59.9
Secondary	47	8.2
House head educational status		
Unable to read and write	88	15.3
Primary school	272	47.4
Secondary school	95	16.6
College /university	119	20.7

Others = Adventist, non-religious

### Prevalence of vernal keratoconjunctivitis

The prevalence of VKC among children in the study area was 64(11.1%), (95% CI: 8, 13.9). Mixed type of VKC was the most prevalent 34(53.1%) followed by palpebral type 28(43.8%)). Forty-four (68.8%) of VKC patients were males giving a male to female ratio of 2.2:1. Fifty percent of VKC cases were in the age group of 5–10 years. Almost all 61(95.3%) of children with VKC had a sign of papillary reaction/cobblestone papillae. All VKC patients had a history of intense itching sensation and two-thirds of them 49(66.2%) have seasonal exacerbations of which 24(49.0%) of them had exacerbations in Spring.

### Factors associated with vernal keratoconjunctivitis

From the bivariate logistic analysis eleven variables namely; sex, age group, educational level of house head, average monthly income, sleeping material, cooking room, having close animal contact and their dander, dust exposure, children systemic allergy history, family systemic allergy history and family ocular allergy history were significantly associated with the occurrence of VKC. Nevertheless, in the multivariable logistic analysis, only 4 variables: male sex, dust exposure, close animal contact, and their dander and systemic (non-ocular) allergy history of a child were independently associated with VKC.

Children who were being males were 4.23 times more to develop VKC as compared to females (AOR = 4.23 (95% CI: 1.33, 13.43)). Those children who were exposed to dust were 4.12 times more to develop VKC than those children who were not exposed (AOR = 4.12 (95% CI: 1.42, 11.91)). In addition, those children who had close animal and their dander contact were 3.38 times more to develop VKC than those who were not exposed (AOR = 3.38 (95% CI:1.14, 10.04)). Similarly, children with a positive history of personal non-ocular allergy were 4.82 times more chance to develop VKC than participants without a history of allergy (AOR = 4.82 (1.40, 16.72)) (Table 2).

**Table 2:** Bivariable and multivariable logistic regression analysis to determine factors associated with vernal keratoconjunctivitis among children living in Gambella town, Southwest Ethiopia (n = 574), June 2018.

Variables	VKC				
	Yes	No	COR (95% CI)	AOR (95% CI)	p-value
Sex					
** Female**	20	235	1.00		
** Male**	44	275	1.88 (1.08, 3.28)	**4.23 (1.33, 13.43)**	0.014^*^
**Cooking room**					
Separated/kitchen	43	398	1.00		
Open field	21	112	1.74(1.00, 3.04)		0.386
Dust exposure					
** No**	38	405	1.00	1.00	
** Yes**	26	105	2.64 (1.53, 4.54)	**4.12(1.42, 11.91)**	0.009^*^
**Sleeping material**					
Foam	55	463	1.00		
Hay	9	47	11.61(0.75, 3.47)		0.699
Animal contact					
** No**	15	181	1.00		
** Yes**	12	21	6.89(2.85, 16.68)	**3.38(1.14, 10.04)**	0.029^*^
**Age group**					
** 2–5 years**	10	93	1.00		
** 6–10 years**	32	189	1.57(0.74, 3.34)		0.633
** 11–18 years**	22	228	0.62(0.38, 1.03)		0.866
**Educational level of house head**					
Unable to write &read	10	78	0.77 (0.33, 1.77)		0.280
Primary school	27	245	0.66 (0.35, 1.27)		0.122
Secondary school	10	85	0.70 (0.31, 1.62)		0.630
College/university	17	102	1.00		
Systemic allergy history					
No	33	485	1.00	1.00	
Yes	31	52	8.27(4.69, 14.60)	**4.82(1.40, 16.72)**	0.013^*^
Family ocular history					
No	53	474	1.00		
Yes	11	36	2.73(1.31, 5.68)		**0.200**
Family systemic allergy					
No	52	486	1.00		
Yes	12	24	4.67 (2.21,9.89)		**0.322**
**Income level**					
≤ 1575	20	123	1.31 (0.64, 2.65)		**0.377**
1576–2400	20	131	1.23 (0.60, 2.51)		**0.957**
2401–3500	9	135	0.53 (0.23, 1.27)		**0.296**
>3500	15	121	1.00		

* P-value <0.05

COR = Crude odds ratio, AOR = Adjusted odds ratio

## Discussion

This community-based cross-sectional study was conducted to determine the prevalence of vernal keratoconjunctivitis and its associated factor among children in Gambella town.

In this study, the prevalence of VKC among children was 64 (11.1%) (95% CI: 8.7, 13.9). This result is higher than school-based study done in Butajira, Southern Ethiopia (5.2%)[17], community based study done in Gondar, Northwest Ethiopia (5.8%)[16], School-based study done in Rwanda (4%)[26], School-based study done in Egypt (3.3%)[33], hospital-based study done in Japan (3.8%)[29] and hospital-based study done in Italy (6.5%)[34]. This might be due to the area of this study was hot, dry and windy which have a greater contribution to allergic diseases of the eye including VKC [6,35]. The other possible justification could due to the difference in study subjects included and methods used by these different studies. In studies done in Japan and Italy cases were detected among all age patients which includes age groups above 20 years who were at low risk for VKC. Studies done in Rwanda and Egypt includes both temperate and hot study areas, however our study had only the hot part of the region that might increase the proportion of VKC.

On the other hand, the prevalence of VKC in this study was lower than studies done in Nigeria (18.1%)[19], Mali (37.2%)[15] and India (18%)[32]. The possible discrepancies could be due to different environmental, hormonal and hereditary factors that contribute to the occurrence of VKC [25,36]. The other possible reason for the difference could also be due to differences in age groups included in the studies. The previous studies included ages below 15 years which was closest to highest risk group of VKC which is manifested in the first decade of life [22,37] whereas our study includes ages till 18 years where VKC is in progress of ceasing. Additional evidence for the lower prevalence of VKC in this study might be all the study (Nigeria, Mali, India) were an institution based, especially a study done in Mali and India were hospital-based which might give high chance for VKC cases.

This finding was supported by other study done in Jerusalem, Palestine (9.8%)[38]. This could be due to that both areas were dry, hot and dusty which might be a contributing factor to allergen/IGE molecules adherence to the eye and adnexa[6].

The proportion of different VKC types varies widely in different studies. In this paper more than half 34 (53.1%) of VKC patients had MVKC, followed by palpebral 28 (43.8%). This finding is supported by other studies done in Ethiopia 35 (81.4%)[16], Egypt (69.80%)[33] and India 102(40.80%)[32]. Other studies including Italy (53.8%)[29], Rwanda (98.4%)[26] and Butajira, Ethiopia (58.5%)[17] reported that limbal type was the most common type of VKC. On the other hand, studies done in Mali (65.22%)[15] and Nigeria (47.1%)[19] had palpebral type VKC in a leading position. Even though, it was not clear why some forms of VKC common in some areas while others not [25], these discrepancies might probably due to different hormonal and hereditary factors[25,36]. Different in setting up, study design and study population might also contribute to the specific type of VKC. Another, possible reason why mixed type was common in our study could be, without any intervention mechanisms, VKC may progress through time to both tarsal and palpebral type[8].

The results of this study showed that the odds of developing VKC among males were 4.23 times more as compared to females (AOR = 4.23, (95% CI, 1.33, 13.43)). This was supported by studies done in Rwanda and Italy [26,30]. Studies showed that male children in dry and hot climate are more affected by VKC as compared to temperate[9] which is similar to our study area. This might due to hormonal factors in the development of VKC. Research has shown that estrogen and progesterone can be detected in the receptors within the epithelium and subepithelium of both the tarsal and bulbar conjunctiva in patients with VKC but not in healthy individuals[39]; this shows an immunoreactivity component to VKC associated with hormonal factors In addition to the above explanation VKC patients have different circulating sex hormone levels relative to nonallergic subjects which suggest a role of sex hormones in the pathogenesis of VKC[40]. Even though the pathogenesis how pollens, grasses, wind exposure, sunlight exposure, and others cause of VKC is not well known, it is common that males are highly prone to these conditions since they spend much of their time in outdoor activities[41]. This could cause the above sources of allergens to reach their eyes that might lead to hypersensitivity reactions to these antigens which in turn lead to VKC[8,25].

The odds of developing VKC among children having a close animal contact in this study were 3.38 times as compared to those not exposed. This might due to animals and their dander have a high probability of harboring different allergen sources which can lead to type I hypersensitivity reaction[25] and pet allergens suspend in the air, stick to different furniture, clothes, walls and other significant areas where children usually spend their time. In addition, children might directly have close contact with these domestic/pet animals which in turn expose them to allergens that might cause a reaction to conjunctiva[42]. A study done in Rwanda showed that those children having close contact to the animals and their dander was less probable to develop VKC (protective factor) as compared to those who do not have contact to the animals[26]. This might be due to the type of animals present and extent of exposure to these animals.

As clearly stated, more than one-third of VKC patients had also multiple types of atopic diseases [22]. Children who had a history of personal systemic (non-ocular) allergy in this study were 4.82 times more probable to acquire VKC as compared to other children with no systemic allergy. This is in consonance with other studies done in Ethiopia[16], Nigeria[4], England[36] and Italy[34]. This might due to the fact that patients with systemic allergic diseases like asthma, bronchitis, eczema and hay fever have similar immunopathology with VKC[6] which carried out by adhering of IgE molecules on the surfaces of mast cells which in turn release inflammation mediators like prostaglandins in the conjunctiva that probably lead to VKC[1,5].

Similarly, those children who were exposed to dust particles were 4.31 times more to develop VKC as compared to non-exposed. This finding is consistent with other studies done in Gondar, Ethiopia, and Rwanda[16,26]. The possible justification might be dust particles especially in the dry and hot season have a greater capability of harboring inflammatory particles[6] which have a higher chance to reach our eyes and develop conjunctival inflammation. In addition, children in this age goups had a high chance to spend their time in dusty areas which are sources of different allergens.

This study determined the prevalence of vernal keratoconjunctivitis as 11.1% which is higher in comparison with studies done in Ethiopia and sub-Saharan countries. This study implies that VKC is a major public health problem which needs screening and greater intervention programmes to upset its blinding complication.

## Conclusion

The prevalence of vernal keratoconjunctivitis among children in Gambella town was high which indicates that it was a public health significance in the town.

Mixed type of vernal keratoconjunctivitis was most common among children in Gambella town.

Sex being male, close animal contact, personal systemic allergy history, and dust exposure were positively associated with vernal keratoconjunctivitis independently.

## Supporting information

S1 File Questionnaire and data extraction form to study prevalence of vernal keratoconjunctivitis and its associated factors among children in Gambella town, southwest Ethiopia, June 2018. (DOCX)

S1 Data Original data. (SAV)
