# Supplementary material for: Prevalence of vernal keratoconjunctivitis and its associated factors among children in Gambella town, southwest Ethiopia, June 2018
Source: PLoS One. 2019 Apr 18;14(4):e0215528. doi: 10.1371/journal.pone.0215528 (PMC6472775; doi:10.1371/journal.pone.0215528)
Supplement: S1 File — (DOCX) [file pone.0215528.s001.docx]

S1 File. Questionnaire and data extraction form to study prevalence of vernal keratoconjunctivitis and its associated factors among children in Gambella town, southwest Ethiopia, June 2018

Amharic version of semi-structured questionnaire

የጎንደር ዩኒቨርሲቲ

**ህክምናና ጤና ሳይንስ ኮሌጅ**

**የዐይን ህክምና ክፍል** መለያ ቁጥር--------------------

የቀበሌ ስም---------------------

**የመጠይቅና ምርመራ ቅጽ**

ጤና ይስጥልኝ ----------------እባላለሁ፡፡ የጋምቤላ ሆስፒታል ሰራተኛና የጥናት ቡድን አባል ነኝ፡፡ በህጻናት ላይ በብዛት የሚከሰተዉን የአይን አለርጅ/መቆጣት በሽታ መጠንና ተያያዥ ምክናያቶች በጋምቤላ ከተማ ዉስጥ በሚገኙ ህጻናት መካከል ቃለ-መጠይቅና ምርመራ በማድረግ እያጣናን እንገኛለን፡፡ይህ ጥናት እርስዎ በሚሰጡን መረጃ ላይ የተመሰረተ ስለሆነ ፍቃድዎ ከሆነ መረጃዉን በመስጠትና ልጅዎን ለአይን ምርመራ እንዲፈቅዱልን በትህትና እንጠይቃለን፡፡ በጥናቱ ላይ መሳተፍ ካልፈለጉ አሁንም ሆነ በሂደቱ ዉስጥ አለመስማማት የችላሉ፡፡ ስለሆነም ጥናቱ ጥቂት ጊዜ ከመዉሰዱ ዉጭ ምንም አይነት ጉዳት የማያስከትል ስለሆንነ እርስዎም ሆነ ልጅዎ እንዲሳተፉ እናበረታታለን፡፡

መረጃዎ ሚስጥራዊነቱ የተጠበቀ ፣ ለጥናቱ አላማ ብቻ የሚዉል ና ለሌላ ጉዳይ የማንጠቀምበት መሆኑን እንገልጣለን፡፡ ቃለ-መጠይቁና ምርመራዉ 15 ደቂቃ ስለሚዎስድ ፈቃደኝነትዎን በፊርማ እንዲያረጋግጡልን በትህትና እየጠየቅን ወደ ቃለ-መጠይቁና ምርመራዉ እናመራለን፡፡

የወላጅ/አሳዳጊ ስም ----------------------------- ፊርማ---------------------ቀን-------------

ለመሳተፍ ፈቃደኛ ከሆኑ ወደ ሚቀጥለዉ ገጽ እናልፋለን፡፡

ማንኛዉም የሚያንሱት ጥያቄ ካለዎተ ተመራማሪዉን በሚቀጥለዉ አድራሻ ማግኘት ይችላሉ፡፡

**ስም** ፡ሶፎንያስ አዲስ **ስልክ**፡ 0918541831

**የመረጃ ሰብሳቢዉ**

ስም----------------------------ፊርማ----------------------------ቀን------------

**መረጃዉን ያረጋገጠዉ ሰዉ**

ስም ----------------------------ፊርማ ---------------------------ቀን-------------

| **ሀ. ማህበራዊ መረጃ** | | | |
| --- | --- | --- | --- |
| ተ.ቁ | ጥያቄዎች | የመልስ አማራጭ | ምርመራ |
|  | መለያ ኮድ |  |  |
|  | እድሜ |  | በአመት |
|  | ጾታ | ሀ. ወንድ ለ. ሴት |  |
|  | ሀይማኖት | ሀ. ኦርቶዶክስ  ለ. ሙስሊም  ሐ. ፕሮቴስታንት  መ. ካቶሊክ  ሰ. ሌላ……………. |  |
|  | ብሔር | ሀ. ኦሮሞ  ለ.ጋምቤ-ህዝቦች (አኙሕ፣ኑዌር፣መጀንግ)  ሐ. አማራ  መ. ትግሬ  ሰ. የደቡብ ብሔር ብሔረሰቦች  ረ. ሌላ………… |  |
|  | የህጻኑ የትምርት ደረጃ | ሀ. ትምህርት ያልጀመረ  ለ. መዋዕለ ህጻናት  ሐ. አንደኛ ደረጃ  መ. ሁለተኛ ደረጃ |  |
|  | የቤተሰብ አስተዳዳሪ የትምህርት ደረጃ | ሀ. ማንበብና መጻፍ የማይችል/የማትችል  ለ. ማንበብና መጻፍ የሚችል/የምትችል  ሐ. አንደኛ ደረጃ  መ. ሁለተኛ ደረጃ  ሰ. ኮሌጅ/ዩኒቨርሲቲ |  |
| **ለ. የማህበራዊ እና ምጣኔ ሀብት ሁኔታ** | | | |
|  | አማካኝ የወር ገቢ መጠን |  | በብር |
|  | የህጻኑ ምኝታ ፍራሽ ምን አይነት ነዉ? | ሀ.ስፖንጅ ለ. ገለባ ሐ. ጥጥ |  |
|  | የመኖሪያ ቤትዎ ወለል ከምን የተሰራ ነዉ? | ሀ.ሸክላ/ሲሚንቶ  ለ. አፈር/መሬት |  |
|  | ምግብ የሚያበስሉት በምንድን ነዉ? | ሀ.በእንጨት/ከሰል/  ለ. በመብራት ሀይል  ሐ. በሁሉም |  |
|  | ምግብ የሚያበስሉት የት ነዉ? | ሀ. የተለየ ቤት/ኩሽና  ለ. ከመኖሪያ ቤት ዉስጥ  ሐ. ከመኖሪያ ቤት ዉጭ እነዲሁ |  |
|  | መኖሪያ ቤትዎ በዉስጡ ሥንት ክፍሎች አሉት? | ----------- |  |
|  | ከቤትዎ አቀራቢያ የሚገኘዉ ዋና መንገድ ምን አይነት ነዉ? | ሀ. አስፓልት/ኮብል  ለ. ኮረኮንች/ጠጠረ  ሐ. አቧራማ አፈር |  |
|  | ሕጻኑ/ኗ ፊት/ሰዉነት ከመታጠብ በኋላ የፊት ማዳረቂያ ፎጣ የመጠቀም ልምድ አለዉ/ላት? | u. አዎ  ለ. የለም | አዎ የምንለዉ ቢያንስ በቀን አነዴ ሲጠቀሙ ነዉ |
|  | በቤት ወይም በቅርብ ጎረቤት የቤት እንስሳቶች ይኖራሉ? | ሀ.አዎ  ለ. አይኖሩም | ምልሱ አይኖሩም ከሆነ ወደ ጥያቄ ቁጥር 17 ይለፉ |
|  | አዎ ካሉ ህጻኑ/ኗ ከእንስሳቶች ወይንም ከጽዳጃቸዉ ጋር ንክኪ የመፍጠር ልምድ አለዉ/ላት? | ሀ. አዎ  ለ.የለም |  |

|  | ባለፉት ስድስት ወራት ዉስጥ ህጻኑ ለአቧራ ተጋልጦ/ጣ ያዉቃል/ታዉቃለች? | ሀ. አዎ  ለ. የለም | አዎ ምንለዉ በትንሹ የአንድ ጊዜ መጋለጥ እና የአይን መቆርቆር/ማሳከክ/ማቀጠል ስሜት ካመጣ ነዉ |  |  |  |  |
| --- | --- | --- | --- | --- | --- | --- | --- |
| **ሐ.የአይን መቆጣት በሽታን በተመለከተ** | | | |  |  |  |  |
|  | ባለፉት ስድስት ወራት ዉሲጥ ህፃኑን የአይን ማሳከክ ተሰምቶት/ቷት ያዉቃል? | ሀ. አዎ ለ. የለም | መልሱ የለም ከሆነ ወደ ጥያቄ 22 ይለፉ |  |  |  |  |
|  | መልሱ አዎ ከሆነ በተጨማሪ ከዚህ በታች ከተዘረዘሩት ስሜቶች የትኛዉ ተሰምቶት/ቷትያዉቃል? | ሀ. የዐይን መቅላት  ለ. ጸሐይ የመፍራት ሁኔታ  ሐ. ወፈር ያለ ቅምጥ  መ. የመቆርቆር ስሜት  ሰ.የማቃጠል ስሜት  ረ.ሌላ ካለ ይጥቀሱ………… |  |  |  |  |  |
|  | ማሳከክ የጀመረበት አመት ስንት ነዉ | ------------ |  |  |  |  |  |
|  | የመቆጣት በሽታዉ የሚበባስበት ወቅት አለ? | ሀ. አዎ ለ.የለም |  |  |  |  |  |
|  | መልሱ አዎ ከሆነ የሚበባሰዉ በትኛዉ የአመቱ ወቅት ነዉ? | ሀ. ጸደይ  ለ. በጋ  ሐ.በልግ  መ. ክረምት |  |  |  |  |  |
|  | ለአይን መቆጣት በሽታ ህክመና ወስደዉ ያዉቃሉ ወይ | 1. አዎ  2. አላዉቅም |  |  |  |  |  |
|  | ህፃኑ/ኗ አጠቃላይ የሰዉነት መቆጣት ምልክት አለበት/ባት? | ሀ. አዎ  ለ. የለም | በማየትም ጭምር ይመለሳል |  |  |  |  |
|  | መልሱ አዎ ከሆነ የትኛዉ አይነት የመቆጣት በሽታ ተከሰተበት/ባት? | ሀ. አስም  ለ. የጉሮሮ ማቃጠል/መቁሰል  ሐ. የቆዳ አለርጂ/መቆጣት  መ.ሌላ………. |  |  |  |  |  |
|  | ከቤተሰብ ዉሰጥ የአይን መቆጣት በሽታ ያለዉ አለ? | ሀ. አዎ ለ. የለም |  |  |  |  |  |
|  | ከቤተሰብ ዉስጥአጠቃላይ የሰዉነት መቆጣት በሸታ ያለዉ ሰዉ አለ? | ሀ. አዎ ለ. የለም |  |  |  |  |  |
|  | መልሱ አዎ ከሆነ የትኛዉ አይነት የመቆጣት በሽታ ተከሰተ? | ሀ. አስም  ለ. የጉሮሮ ማቃጠል/መቁሰል  ሐ. የቆዳ አለርጂ/መቆጣት  መ.ሌላ………. |  |  |  |  |  |
|  | **physical examination** | |  |  |  |  |  |
|  |  | Anterior segment examination | RE | LE |  |  |  |
|  |  | 1.tarsal conjunctiva | yes | No | Yes | No |  |
|  |  | Papillary reaction/cobblestone papillae |  |  |  |  |  |
|  |  | Mucoid discharge |  |  |  |  |  |
|  |  | Others |  |  |  |  |  |
|  |  | Normal |  |  |  |  |  |
|  |  | 2.bulbar conjunctiva |  |  |  |  |  |
|  |  | Redness |  |  |  |  |  |
|  |  | swelling |  |  |  |  |  |
|  |  | Other |  |  |  |  |  |
|  |  | Normal |  |  |  |  |  |
|  |  | 3.limbus |  |  |  |  |  |
|  |  | Opacifications |  |  |  |  |  |
|  |  | Thickening |  |  |  |  |  |
|  |  | Widening |  |  |  |  |  |
|  |  | Horner Trantas Dot |  |  |  |  |  |
|  |  | Others |  |  |  |  |  |
|  |  | Normal |  |  |  |  |  |
|  |  | 4.cornea |  |  |  |  |  |
|  |  | Superior pannus |  |  |  |  |  |
|  |  | Superficial keratitis |  |  |  |  |  |
|  |  | Suspected shield ulcer |  |  |  |  |  |
|  |  | Others |  |  |  |  |  |
|  |  | Normal |  |  |  |  |  |
|  |  | 5.final assessment |  |  |  |  |  |
|  |  | VKC |  |  |  |  |  |
|  |  | Other eye diseases |  |  |  |  |  |
|  |  | Normal |  |  |  |  |  |
|  |  | 6.if VKC, specify the type of VKC |  |  |  |  |  |
|  |  | Palpebral type |  |  |  |  |  |
|  |  | Bulbar type |  |  |  |  |  |
|  |  | Mixed type |  |  |  |  |  |

**English version of structured questionnaire**

Semi-structured questionnaire with data extraction form for assessment of prevalence of vernal keratoconjunctivitis and its associated factors among children living in Gambella town, Southwest Ethiopia.

**Introduction**

Good morning/afternoon, my name is --------------------------. I am working for Gambella hospital and I am a member of a research group working in Gambella. I am studying the prevalence of vernal keratoconjunctivitis and its associated factors among children in Gambella town by asking questions and physical examinations. Your answers for all of our questions are important to determine prevalence of vernal keratoconjunctivitis and its associated factors. Your answers will be secret and confidential. If you decide not to participate in the study now or at any time in the progress of the study you can withdraw from the study. But we appreciate you if you try to participate and we use 15 minutes to complete the questionnaire and physical examination. Thank you. Next, I will read a consent which assures your interest to participate.

Do I have your permission to continue?

If yes thank you and continue.

If no thank you and go to next study subject.

**Data collector**

Name ------------------------------signature --------------------------date ----------------

**Checked by supervisor**

Name--------------------------------signature---------------------------date------------------

| **A**. **demographic conditions** | | | | | | |
| --- | --- | --- | --- | --- | --- | --- |
| S. N | Questions | Responses/answers | | Remarks | | |
|  | ID |  | |  | | |
|  | Age |  | | In year | | |
|  | Sex | 1. Male 2. female | |  | | |
|  | Religion | 1. Orthodox 2. Muslim 3. Protestant 4. Catholic 5. Others…... | |  | | |
|  | Ethnicity | 1. Oromo 2. Gambella (Anuak, Nuar Mejeng ) 3. Amhara 4. Tigray 5. South nations and nationalities 6. Others……… | |  | | |
|  | Educational status of a child | 1.no schooling  2.kindergarten/KG  3. primary school  4. secondary school | |  | | |
|  | Educational status of household head | 1. Unable to write and read 2. Able to write and read 3. Primary school 4. Secondary school 5. College/university | |  | | |
| **B. Socio-economic characteristics** | | | | | | |
|  | Monthly income |  | | In birr | | |
|  | Sleeping materials | 1. Foam mattress 2. Hay mattress 3. Cotton mattress | |  | | |
|  | House floor condition | 1. Cemented/clay 2. Earth | |  | | |
|  | Cooking materials | 1. Fire wood/kerosene 2. Electric energy 3. Both | |  | | |
|  | Cooking room condition | 1. Within living room 2. Separated room 3. Open field | |  | | |
|  | Number of rooms in the house |  | |  | | |
|  | Type of main road near by the house | 1. Asphalt 2. beaten path 3. dirt soil road | |  | | |
|  | Usage of face towel after face washing | 1. Yes 2. No | | Usage at least once a day is taken as yes | | |
|  | Presence of any domestic animals in the house or neighbourhoods | 1. Yes 2. No | | If yes continue the question if no pass to question 17 | | |
|  | If yes, did the child has any contact to the animals or their dander in the last 7 days and followed with ocular irritation | 1. Yes 2. No | |  | | |
|  | Dust exposure in the last 6 months | 1. Yes 2. No | | At least one episode of exposure to the dust into either eye and causing ocular irritation | | |
| **C. vernal keratoconjunctivitis status** | | | | | | |
|  | Did you experience intense itching sensation within the last 6 months in either eye | 1. Yes 2. No | | If yes continue to the next, if no skip to question 19 | | |
|  | If yes which additional symptoms did you experience from the following list | 1. Tearing 2. Photophobia 3. Tearing 4. Redness 5. Burning sensation 6. Foreign body sensation 7. Others | | If the answer is the other go to question 20 | | |
|  | Age of onset for itching in year | ------------ | |  | | |
|  | Seasonal variation | 1. Yes 2. no | | For the selected symptoms | | |
|  | If yes when was become more sever | 1. autumn 2. winter 3. spring 4. summer | |  | | |
|  | Any history of treatment for allergic eye diseases | 1. yes 2. no | |  | | |
|  | Any associated systemic allergic history | 1. yes 2. no | |  | | |
|  | If yes which from the following list | 1. asthma 2. bronchitis 3. atopic dermatitis 4. other | |  | | |
|  | Any family ocular allergic history | 1. yes 2. no | |  | | |
|  | Any family systemic allergic history | 1. yes 2. no | |  | | |
|  | If yes which one from the following list | 1. asthma 2. bronchitis 3. atopic dermatitis 4. others | |  | | |
| **physical examination** | | | | | | |
|  | Anterior segment examination | RE | | LE |  | |
|  | 1.tarsal conjunctiva | yes | No | Yes | No |  |
|  | Papillary reaction/cobblestone papillae |  |  |  |  |  |
|  | Mucoid discharge |  |  |  |  |  |
|  | Others |  |  |  |  |  |
|  | Normal |  |  |  |  |  |
|  | 2.bulbar conjunctiva |  |  |  |  |  |
|  | Redness |  |  |  |  |  |
|  | swelling |  |  |  |  |  |
|  | Other |  |  |  |  |  |
|  | Normal |  |  |  |  |  |
|  | 3.limbus |  |  |  |  |  |
|  | Opacifications |  |  |  |  |  |
|  | Thickening |  |  |  |  |  |
|  | Widening |  |  |  |  |  |
|  | Horner Trantas Dot |  |  |  |  |  |
|  | Others |  |  |  |  |  |
|  | Normal |  |  |  |  |  |
|  | 4.cornea |  |  |  |  |  |
|  | Superior pannus |  |  |  |  |  |
|  | Superficial keratitis |  |  |  |  |  |
|  | Suspected shield ulcer |  |  |  |  |  |
|  | Others |  |  |  |  |  |
|  | Normal |  |  |  |  |  |
|  | 5.final assessment |  |  |  |  |  |
|  | VKC |  |  |  |  |  |
|  | Other eye diseases |  |  |  |  |  |
|  | Normal |  |  |  |  |  |
|  | 6.if VKC, specify the type of VKC |  |  |  |  |  |
|  | Palpebral type |  |  |  |  |  |
|  | Bulbar type |  |  |  |  |  |
|  | Mixed type |  |  |  |  |  |
